# Epigenetic alterations in ameloblastomas: A literature review

**DOI:** 10.4317/jced.56191

**Published:** 2021-03-01

**Authors:** Erison-Santana Santos, Carla-Isabelly Rodrigues-Fernandes, Joab-Cabral Cabral, Felipe-Paiva Fonseca, Adriana-Franco-Paes Leme

**Affiliations:** 1 Department of Oral Diagnosis, Piracicaba Dental School, University of Campinas, Piracicaba, Brazil; 2 Department of Oral Surgery and Pathology, School of Dentistry, Federal University of Minas Gerais, Belo Horizonte, MG, Brazil; 3 Brazilian Biosciences National Laboratory, The Brazilian Center for Research in Energy and Materials, Campinas, Brazil

## Abstract

**Background:**

Ameloblastoma is a locally aggressive tumor, originated from odontogenic epithelium, and affects the jawbones with an elevated recurrence rate. The molecular mechanisms involved with the pathogenesis of this tumor remain undetermined. This review aimed to describe the current data regarding epigenetic alterations in ameloblastoma.

**Material and Methods:**

A systematized electronic search was performed in the English-language literature in three databases, combining the following keywords: ameloblastoma, epigenetic, methylation, noncoding RNA, histone acetylation.

**Results:**

According to the gathered results of 11 studies in this review, epigenetic alterations could induce the development and progression of ameloblastoma. DNA methylation has been the most assessed mechanism in ameloblastomas.

**Conclusions:**

Current literature data indicate that epigenetic events can be involved in the etiopathogenesis of ameloblastomas.

** Key words:**Ameloblastoma, epigenetic, methylation, noncoding RNA, histone acetylation.

## Introduction

Gene mutation has been considered the leading cause of tumorigenesis, generating permanent modifications in oncogenes and tumor suppressor genes. These modifications transform proteins’ production and expression, which cause dysregulation of cell-cycle control, leading to uncontrolled cell proliferation and tumor formation (1,2). Similarly, genetic and epigenetic alterations can influence each other, and both play a significant role in the initiation and progression of neoplasms, including odontogenic tumors (2-4).

**Figure 1:**
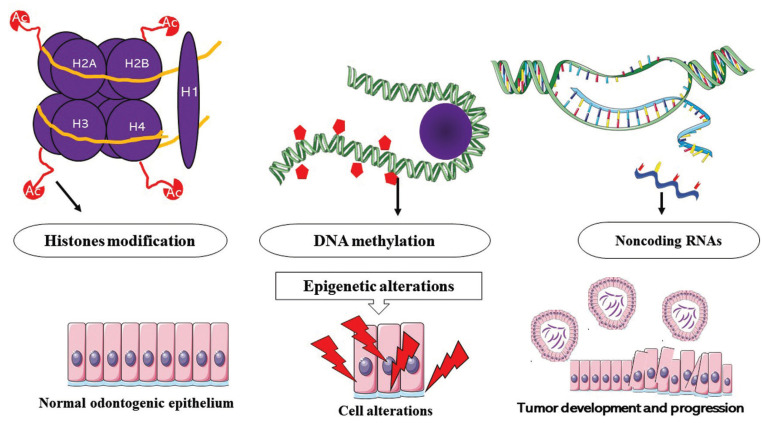
Epigenetic alterations may be involved in the development and progression of tumors. Many histones may present different structural modifications leading to an alteration in the transcription, repair, and replication of DNA. DNA methylation and demethylation are common in ameloblastomas by the addition of methyl groups to CpG islands, and this event causes altered regulation of gene expression. Noncoding RNAs can bind to coding RNAs, such as mRNA, and modify protein expression. All these isolated or combined events gradually contribute to the organization of an unstable genome, and the promotion of tumor development and progression. Probably, if these events occurred in the odontogenic epithelium may cause odontogenic tumors. These epigenetic events are not exclusive of ameloblastomas; they can occur in other benign and malignant tumors.

Epigenetic alteration is defined as a group of modifications, which occur at a genomic level, and do not change the DNA base sequence but alter the DNA conformation and modify the genetic expression (5). In contrast with genetic mutations, epigenetic alterations are dynamic and reversible, indicating a potential therapeutic alternative to several neoplasms (5). The main types of these alterations are DNA methylation, modification on the expression of noncoding RNAs, and conformational modification of histones (Fig. 1) (6). DNA methylation frequently occurs in the fifth position of cytosine (5mC), and it is associated with gene repression and activation, splicing regulation, imprinting, nucleosomes positioning, and recruitment of transcription factors (7). Several enzymes are involved with this event, named as DNA methyltransferases (DNMTs), and mainly comprise DNMT1, DNMT3A, DNMT3B, so as the cofactor DNMT3L. Moreover, a recently fourth DNA methyltransferase was described: DNMT3C (8). DNMTs 3A and 3B are responsible for *de novo* methylation since they are unable to discriminate methylated from unmethylated regions (4,8,9). DNMT1 is essential to preserve the methylation pattern of the template strand for other strips that arise after DNA duplication (9). DNA hyper or hypomethylation may induce the development of tumors by regulating their components, such as the proteins of the extracellular matrix (3).

Changes in noncoding RNAs’ expression are also epigenetic events. The main category comprises micro RNAs (miRNAs), which are synthesized and processed in the nucleus (pre-miR) before exportation to cytoplasm as mature miRNAs (10). They can bind to messenger RNA (mRNA) through complementary sequences and then, may cause post-translational gene silencing by controlling mRNA translation into proteins (11,12). It is likely that the high expression of noncoding RNA leads to a more aggressive phenotype in several neoplasms, including odontogenic tumors (12).

Histones are proteins responsible for DNA condensation, representing a basic component of the nucleosome (5,7,13). These proteins also provide physical support to DNA and play a role regulating its transcription, repair, and replication (13). The structure of histones comprises a flexible “tail,” which is susceptible to post-translation biochemical modifications, such as acetylation, methylation, ubiquitination, and phosphorylation (13). In this context, acetylation and methylation are the most described epigenetic events (13,14). The first reduces the affinity of histones for DNA and creates an “opened” chromatin to enable gene transcription. Histone deacetylation is associated with closed or repressive chromatin, while histone methylation may be associated either with transcriptional activation or repression (13,14).

Ameloblastoma is the most frequent and aggressive intraosseous tumor originated from the odontogenic epithelium, comprising 13–58% of all odontogenic tumors (15,16). It was initially recognized by Cusack in 1827 and described by Broca in 1868 (16). Although ameloblastoma may encompass an extensive age range (8-92 years old), it usually affects patients between the fourth fifth decades of life, with no differences between sexes’ distribution (15). The three distinctive clinicopathological types of ameloblastoma are conventional, unicystic, and peripheral (15).

Despite study advances with regards to the molecular biology of ameloblastoma, its etiology remains unclear. The mutational landscape of odontogenic tumors has not been fully characterized, while some proteins involved in the mitogen-activated protein kinase (MAPK) pathway, such as BRAF, play a pivotal role in the development and progress of ameloblastoma (17). MAPK is active in several biologic processes; however, mutation of their proteins leads to uncontrolled signaling, increasing cell proliferation, survival, and neoplastic transformation (17). BRAF-V600E is the most common mutation, which is associated with clinical and molecular behavior of ameloblastoma. This mutation has also been implicated in both diagnosis and prognosis (17). Additionally, somatic mutations in no-MAPK proteins, such as Smoothened (SMO) may enhance cell proliferation and survival, leading to poor prognosis in ameloblastomas (7,17). SMO is involved in the sonic hedgehog pathway, an essential signaling pathway that impacts tumorigenesis (7,17).

The expression of DNMT’s and DNA methylation of several genes in odontogenic tumors were previously demonstrated (3,18). Similarly, modification of histones and noncoding RNAs have been described in odontogenic tumors, especially ameloblastomas, indicating a potential function of epigenetics in the development and progression of neoplasms (16,19-22).

Although other studies have analyzed the influence of epigenetic events in odontogenetic tumors (7), there is no current data in the literature regarding the association of these events with ameloblastoma. Considering this scenario, the purpose of this study is to provide a systematized literature review on the correlation between epigenetic alterations and the pathogenesis of ameloblastoma.

## Material and Methods

An electronic search without time restriction, but limited to English-language was performed in the following databases: MEDLINE (Medline Industries, Mundelein, Illinois) by PubMed platform (National Center for Biotechnology Information, US National Library of Medicine, Bethesda, Maryland), ScienceDirect and Scopus (Elsevier, Amsterdam, The Netherlands). In the Medline/PubMed database, the following terms were used: “Ameloblastoma” AND “Epigenetic,” “Ameloblastoma,” AND “Methylation” “Ameloblastoma” AND “Noncoding RNA” “Ameloblastoma” AND “Histone Acethyletion.” In both the ScienceDirect and Scopus database, the following terms were used: “Ameloblastoma” AND “Epigenetic.” In order to retrieve as much data as possible, the gray literature (Google Scholar and ProQuest) and was investigated by utilizing the terms: “Ameloblastoma” AND “Epigenetic.” Moreover, a manual search was carried out to identify possible additional studies. The search was updated in august 2019. The articles that lack or did not present relevant information related to the scope of this review were excluded. We also excluded review short communications, encyclopedias, conference abstracts, inaccessible studies online, and articles in different languages than English.

## Results

**Figure 2:**
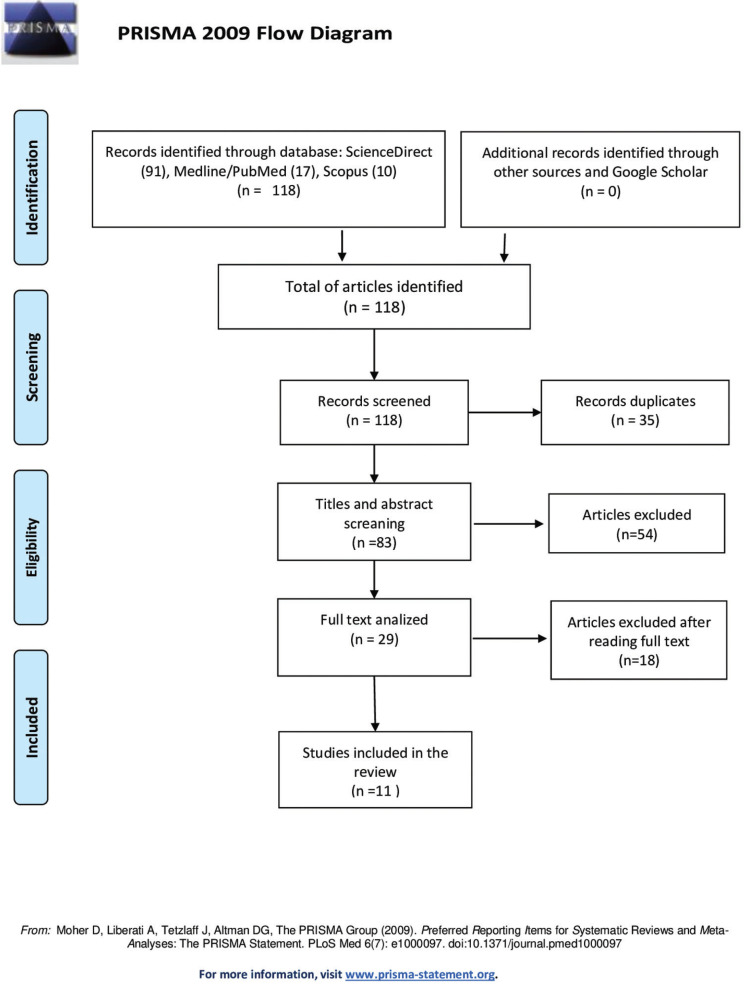
PRISMA flow diagram.

In the first stage of this review, 118 studies were found in the three databases. After duplicate articles were removed, 83 remained. A screening of the titles and abstracts was carried out, and 29 records were included for full-text review. After inclusion and exclusion criteria, a total of 11 articles were selected for data extraction and qualitative synthesis. Figure 2 details this process of study selection. Information about the included manuscripts is summarized in  Table 1 and  Table 2.

**Table 1:**
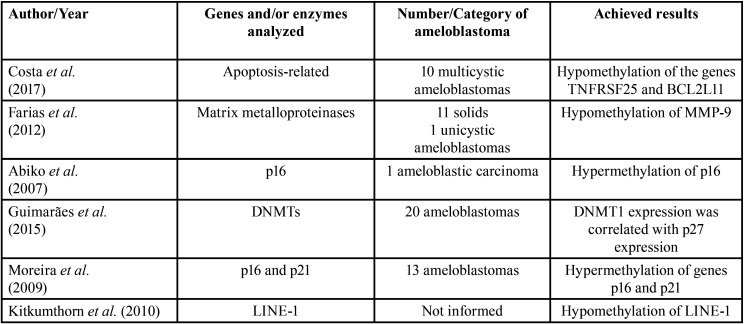
Analyses of DNA methylation and/or enzymes associated with methylation in ameloblastomas published in the literature.

**Table 2:**
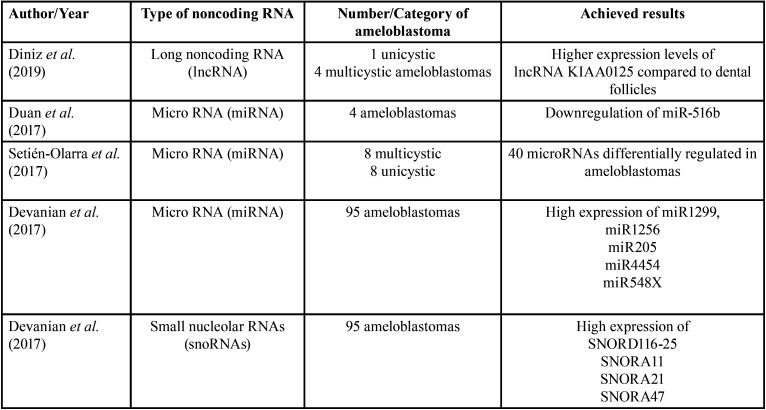
Analyses of noncoding RNAs in ameloblastomas published in the literature.

## Discussion

It is well established that DNA methylation causes gene silencing, leading to the modification of proteins’ expression by blocking transcription after methyl-binding (18). Then, other proteins build a complex which blocks access for transcription factors to the gene promoter (23). Conversely, loss of DNA methylation and/or global hypermethylation is recognized as early events in tumor development, and their manifestation in repetitive regions of the genome results in chromosomal instability (24). DNA methyltransferases (DNMTs) are enzymes that promote either maintenance or *de novo* methylation of DNA (24). It has been demonstrated that the high expression of DNMTs in odontogenic tumors’ cells is an important mechanism for their development, in particular the expression of DNMT1 and 3B (4,9). The expression of DNMT1 in ameloblastomas can deregulate the role of p27 in cellular biological processes (4).

A recent study evaluated the methylation status of 22 apoptotic-related genes in 10 multicystic ameloblastomas. The authors showed that the genes TNFRSF25 and BCL2L11 demonstrated differences in methylation levels compared to dental follicles (14). BCL2L11 is one of the most important regulators of apoptosis, and its transcription is likely to be regulated by promoter DNA methylation in ameloblastomas, which may affect the biologic behavior of this neoplasm (14).

The matrix metalloproteinases (MMPs) are zinc-dependent enzymes involved in extracellular matrix remodeling. They are also correlated with tumor growth and invasion through collagen matrix degradation, as the overexpression of MMP-2 and MMP-9 have been associated with aggressive behavior in ameloblastomas (25,26). The DNA methylation status of these MMPs was investigated in 12 ameloblastomas (11 solid and one unicystic), and in healthy gingival tissue, and the authors indicated that hypomethylation of MMP-9 might be a mechanism involved in the increased transcription of the gene in this tumor (3).

DNA methylation may also be involved with malignant transformation of ameloblastoma since hypermethylation of CpG islands of the p16 gene, which acts as a negative regulator of cellular proliferation, was identified in malignant segments of the tumor (27). This suggests that hypermethylation of p16 and subsequent inactivation may cause deregulation of cell proliferation (27). Nevertheless, the exact function of methylation on cell-cycle genes in ameloblastoma remains unclear. Despite the high profile of methylation p16 and p21 genes observed in this tumor, no significant association was described between the methylation profile of these genes with dental follicles and other epithelial odontogenic tumors (27).

The long interspersed nuclear element-1 (LINE-1 or L1) is a non-LTR (long terminal repeat) retrotransposons, which are widespread in the genome of eukaryotes, corresponding to approximately 17-21% of the human genome (29,30). LINE-1 elements are frequently methylated in normal conditions, and their hypomethylation has been associated with several types of malignancies (29,30). The global profile of hypomethylation in ameloblastomas is similar to head and neck cancers, urinary bladder, liver, gland prostate, and lung cancers, whereas the levels of LINE-1 is significantly reduced in comparison with odontogenic keratocysts (30).

The long noncoding RNAs (lncRNAs) are transcribed RNA molecules containing more than 200 nucleotides, which can affect the expression level of a broad spectrum of genes (12). Different types of noncoding RNAs may contribute to physiologic processes, such as the morphologic development of the tooth and in the etiopathogenesis of malignant tumors, such as pancreatic cancer, colon cancer, and oral squamous cell carcinoma (31-34). Diniz *et al.* compared the expression levels of lncRNA KIAA0125 in dental follicles with ameloblastomas and reported that the lncRNA KIAA0125 is likely to be involved in the pathobiology of this tumor (12). The specific biologic function of lncRNA KIAA0125 remains undetermined, although KIAA0125 is predicted to interact with other noncoding RNAs, such as miRNAs (12).

MicroRNAs are small noncoding RNA molecules, which regulate post-transcriptional gene expression, and have been associated with the etiopathogenesis of ameloblastomas (20). An *in vivo* study evaluated the effects of miR-516b and demonstrated that its overexpression significantly suppressed cell growth and inhibited cell migration and invasion capacity in ameloblastoma cells by inducing cell cycle arrest and apoptosis, suggesting a tumor-suppressive role of this miRNA in ameloblastoma (21). Moreover, the effect of miR-516b in these tumors’ functions occurs through regulation of the c-Myc/RECK/MMPs pathway by inducing MYCBP expression (21). MYCBP, initially identified as an MYC-interacting protein, acts as a positive modulator of the mammalian Hedgehog (Hh) signaling pathway (21,35), which was previously associated with tumorigenesis (35).

**Figure 3:**
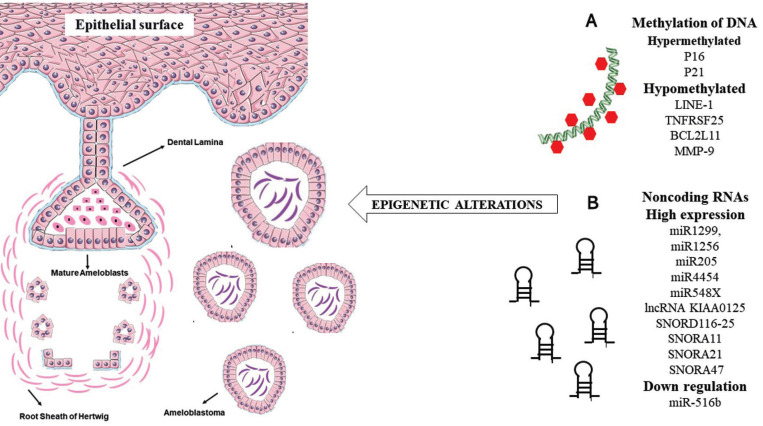
Summary of epigenetic events that can lead to the development and progression of ameloblastomas. (A) Hyper and hypomethylation of genes that were described “in this review”. (B) Noncoding RNAs that have been associated with high expression or downregulation “in ameloblastoma”.

A recent study demonstrated the overexpression of miR1299, miR1256, miR205, miR4454, and miR548X in ameloblastomas by using microarrays. These miRNAs are associate with breast cancer (miR548), bladder tumors (miR454), and prostate cancer (miR1256) (36). The overexpression of miR1299 has not been described in ameloblastomas, but the authors suggested its influence in the etiopathogenesis of this tumor, as well as the use of this noncoding RNA as an excellent tumor marker for ameloblastoma. Nevertheless, validation studies are still necessary (36) (Fig. 3).

The identification of noncoding RNAs may also support the diagnosis and treatment of different lesions, as demonstrated in a study which compared the expression of miRNAs in ameloblastomas and dentigerous cysts and identified 40 microRNAs differently expressed in ameloblastomas. These findings indicated that the differences found in the expression of miR-489 between solid and unicystic subtypes might be useful to elucidate the different patterns of aggressiveness observed in these tumors (20). In addition, the overexpression of miR-31 and miR-135b may be related to neoplastic proliferation and tumor growth, as well as the expression of miR-592 (20).

Small nucleolar RNAs (snoRNAs) comprise a particular group of noncoding RNAs, which role in odontogenic tumors is still unknown, although the overexpression of SNORD116-25, SNORA11, SNORA21, SNORA47 was previously demonstrated in ameloblastomas (36). The high expression of SNORA21 and SNORA47 in patients diagnosed with non-small cell lung cancer has been associated with poor overall survival (37). Likewise, the expression of SNORD116-25 and SNORA65 have been associated with treatment failure of multiple myeloma and ovarian adenocarcinoma (38). Considering that these noncoding RNAs are not easily degraded in body fluids like saliva, they may represent valuable biomarkers (36).

With regards to the expression and modification of histones and histones methyltransferases, previous studies have described these phenomena in oral squamous cell carcinoma and in tooth development (39,40), although investigations on ameloblastomas have not been reported.

Among different epigenetic regulators, it has been shown that polycomb group (PcG) proteins have played an important role in tumor development, throughout the control of cellular proliferation, differentiation, and invasion (19). These complexes modify histones conformation and silence specific sets of targeted genes by altering the structure of chromatin (19). The presence of patterns of five human PcG proteins (Bmi-1, Ring1b, Mel-18, Ezh2, and Suz12) in ameloblastomas and odontogenic keratocysts was previously analyzed. The authors concluded that, according to the location of immunopositive cells, Mel-18 and Ezh2 might be involved in the growth of odontogenic keratocysts, and almost all PcG proteins are possibly associated with cell proliferation and differentiation of ameloblastomas (22).

## Conclusions

Epigenetic modifications are possibly associated with the etiopathogenesis of ameloblastomas, and DNA methylation is the most assessed epigenetic event. Abnormal DNA methylation may lead to dysregulation of the cell cycle and then influence the biological behavior of ameloblastomas. Modifications on the expression of noncoding RNAs may also be involved in the pathobiology of these tumors, despite the paucity of current studies evaluating these events. Then, due to the frequency and aggressiveness of ameloblastomas, this review confirmed the necessity to provide a better comprehension with regards to the role of epigenetic mechanisms in the development of this neoplasm.
